# A Case of Severe Psychosis Due to Cushing's Syndrome Secondary to Primary Bilateral Macronodular Adrenal Hyperplasia

**DOI:** 10.7759/cureus.6162

**Published:** 2019-11-15

**Authors:** Kajal Shah, Inderjit Mann, Kalpana Reddy, Geevarghese John

**Affiliations:** 1 Internal Medicine, Northwell Health-Long Island Jewish Forest Hills Hospital, Forest Hills, USA; 2 Endocrinology, Northwell Health-Long Island Jewish Forest Hills Hospital, Forest Hills, USA

**Keywords:** hypercortisolism, psychosis, ketoconazole, cushing, primary bilateral macronodular adrenal hyperplasia (pbmah)

## Abstract

Hypercortisolism is a multisystem disorder that results from inappropriate and excessive glucocorticoid secretion and loss of normal feedback mechanisms of the hypothalamic-pituitary axis. It is broadly divided into adrenocorticotropic hormone (ACTH) dependent and ACTH-independent categories. Primary bilateral macronodular adrenal hyperplasia (PBMAH) is a rare cause of ACTH-independent hypercortisolism, accounting for less than 2% of cases. It usually presents as hypertension, metabolic abnormalities, thromboembolic, cardiovascular, or endocrine disorders but rarely as a neuropsychiatric illness. Therefore, a delay in the diagnosis and management of cognitive illnesses substantially increases morbidity in these patients. Herein, we report a case of severe psychosis due to Cushing's syndrome with PBMAH.

A 49-year-old male with a past medical history of diabetes and hypertension presented with acute onset of confusion. The patient’s uncontrolled hypertension, hypokalemia, metabolic alkalosis, and resistant psychosis to various psychotropic medications raised the suspicion of an underlying metabolic disorder. Further workup revealed an inappropriate suppression of morning (AM) cortisol after administration of dexamethasone and elevated values of serum AM cortisol and 24-hour urinary cortisol, in addition to low ACTH. Computed tomography (CT) of the abdomen and pelvis with intravenous (IV) contrast was performed to evaluate the adrenal gland which showed multiple nonspecific adrenal nodules bilaterally measuring between 3.5 cm - 4.5 cm. The patient was hence diagnosed with hypercortisolism secondary to PBMAH. The patient was treated with ketoconazole after he refused surgery as a treatment option and was noted to have significant improvement in his psychosis within a week, along with improvement of his hypertension, electrolyte abnormalities, and a significant decrease in the 24-hour urine cortisol level.

Neuropsychiatric illness is a rare manifestation and an unusual initial presenting symptom of Cushing’s syndrome secondary to primary bilateral macronodular adrenal hyperplasia. A delay in diagnosis often subjects these patients to unnecessary psychotropic medications and prolonged psychiatric hospitalizations. Hence, clinicians must be cognizant of this rare entity when making a diagnostic evaluation to prevent subsequent morbidity and mortality.

## Introduction

Endogenous Cushing’s syndrome (CS) is a rare endocrine disease caused by hypercortisolemia with an incidence of 0.7 - 2.4 cases per million annually [1-2]. The etiology of CS may be adrenocorticotropic hormone (ACTH)-dependent (80% - 85%) or ACTH-independent (15% - 20%) [3-4]. ACTH-dependent causes include, most commonly, pituitary adenoma (Cushing’s disease) and, less frequently, ectopic ACTH-secreting malignancies. Unilateral adrenocortical tumors occur in 75% - 95% of patients with ACTH-independent CS, followed by adrenocortical carcinoma in 5% of cases. Primary bilateral macronodular adrenal hyperplasia (BMAH) is an uncommon cause (< 2%) of endogenous ACTH-independent CS presenting mainly as subclinical CS with no clinical signs and symptoms as an incidental finding; however, it can rarely lead to overt CS [5]. Although non-psychiatric clinical features, such as hypertension, metabolic abnormalities, cardiovascular, or endocrine disorders, are more common findings in hospitalized patients with overt CS, psychosis or confusion could also be presenting symptoms [6]. In such cases, if hypercortisolemia is untreated, psychiatric symptoms remain refractory to psychotropic medications and can cause significant mortality and morbidity. Glucocorticoids (GCs) play a vital role in the functioning and homeostasis of the hypothalamic-pituitary-adrenal axis and central nervous system. GC receptors have a pleiotropic distribution in the central nervous system, mainly in the hippocampus and neocortex; therefore, chronic exposure to supraphysiological levels of GC in CS can lead to anatomical and physiological changes in the brain causing psychiatric diseases, cognitive impairment, mood disorders, and sleep disturbances [7-8]. Harvey Cushing was first to describe psychiatric illness in Cushing’s syndrome in 1912 as emotional disturbances [9]. In the following years, several studies were performed to better characterize the spectrum and prevalence of neuropsychiatric disorders in CS. The incidence and prevalence of neuropsychiatric symptoms vary, with depression being the most common symptom, seen in 50% - 70% cases of CS, followed by anxiety (12% - 79%) and hypomania (3%) [10]. Psychosis and mania are uncommon presentations of CS and only a few cases have been reported in the literature so far. We present a case of severe psychosis in a patient with CS from underlying BMAH. 

## Case presentation

**Figure 1:**
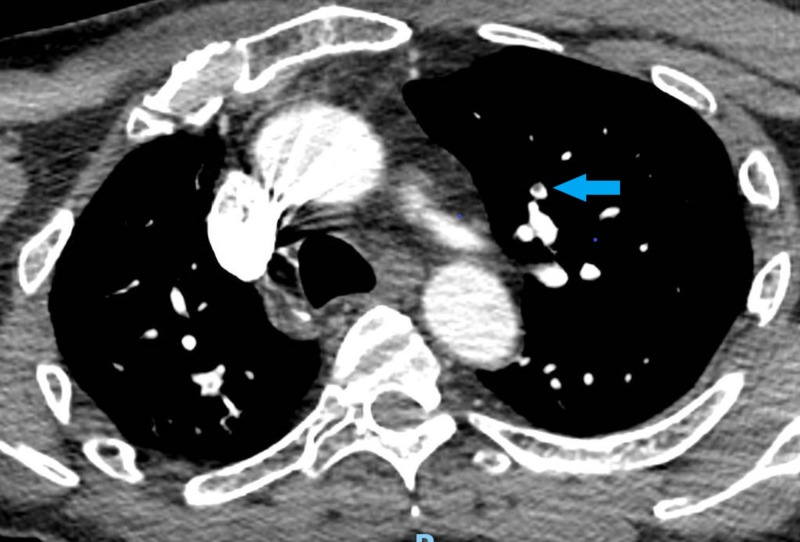
Computed tomography of the chest showing pulmonary embolism

**Figure 2:**
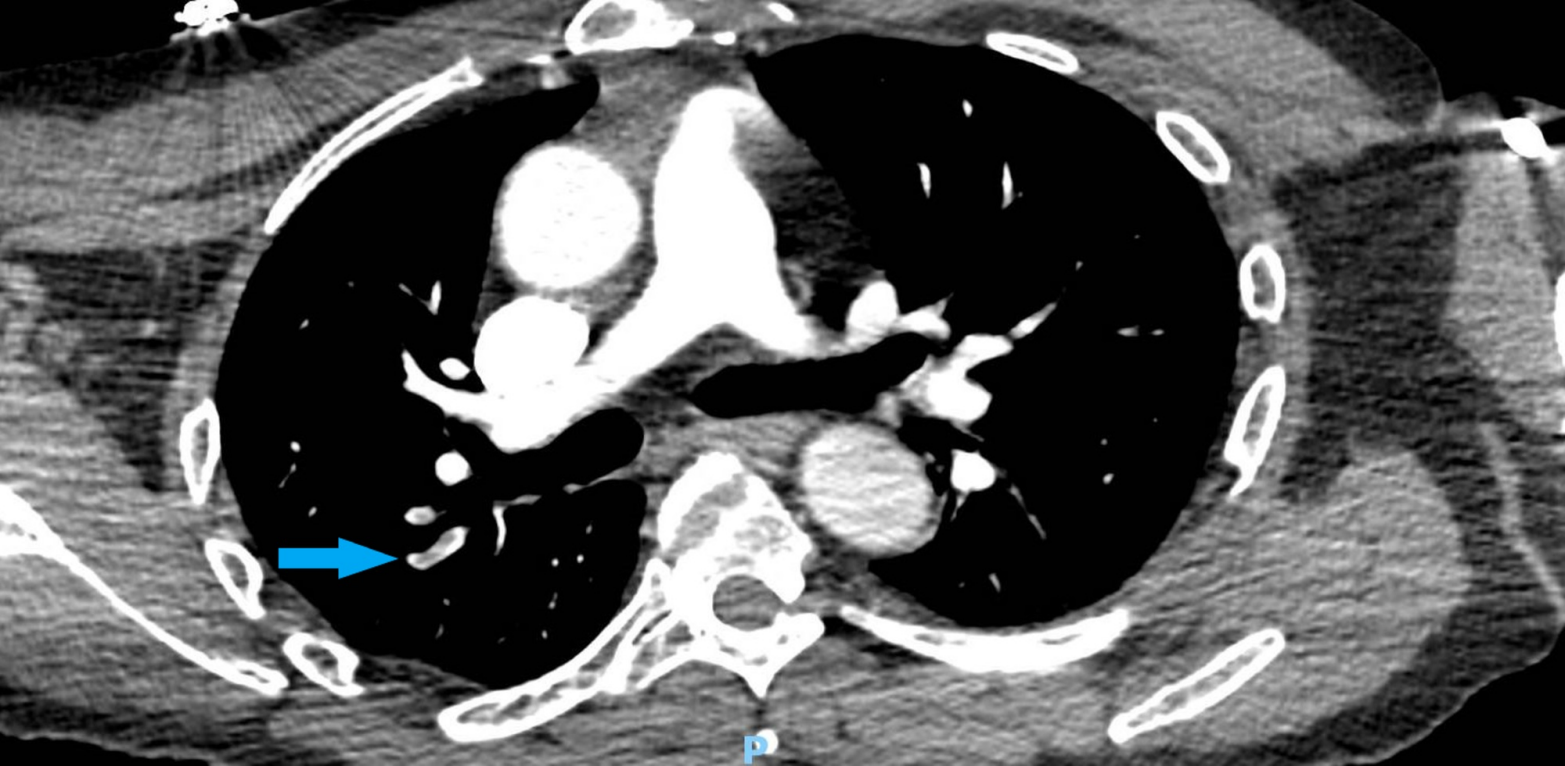
Computed tomography of the chest showing pulmonary embolism

A 49-year-old male presented with left-sided weakness and confusion for two days. His past medical history included diabetes mellitus treated with metformin and hypertension treated with valsartan/hydrochlorothiazide and clonidine. He also had a right knee replacement one week prior to his presentation. Physical examination showed a well-appearing man, who was oriented to only place and person and was noted to have confabulations and illogical thoughts, in addition to visual and auditory hallucinations. On admission, the patient was afebrile with a blood pressure of 167/100 mm hg, a heart rate of 134, a respiratory rate of 17 breaths per minute, and oxygen saturation of 100% on ambient air. His initial electrocardiogram (EKG) showed sinus tachycardia. The patient was admitted for further evaluation of his encephalopathy. His initial laboratory tests were significant for a potassium of 2.7 (normal: 3.5 - 5.3 mmol/L), serum bicarbonate of 35 (normal: 22 - 31 mmol/L), blood glucose of 183 (normal: 70 - 99 mg/dl), thyroid-stimulating hormone (TSH) of 1.28 (normal: 0.34 - 4.82 microunits/mL), and hemoglobin A1c (HbA1c) of 8.1%. Further workup was done to rule out other causes of the encephalopathy which were all negative, including urine toxicology, chest x-ray, computed tomography (CT) scan, magnetic resonance imaging (MRI) of the head, and an electroencephalogram. However, given his sinus tachycardia and confusion, a pulmonary embolism was considered as a differential, which was confirmed on a CT scan of the chest (Figures 1-2) and appropriate treatment was started. Despite the treatment of underlying causes, the patient remained agitated, confused, and paranoid. The patient was seen by a psychiatrist for his symptoms, who suggested an initial therapy with haloperidol, 1 mg twice daily, and lorazepam as required for agitation, along with constant observation for patient safety. Due to the lack of desired clinical improvement over the next few days, other antipsychotics, including risperidone and citalopram, were added. However, those measures failed to control his episodes of severe psychosis. The patient’s hospital stay was further complicated by uncontrolled hypertension despite being on four antihypertensive medications of different classes at their maximum doses (valsartan, hydralazine, labetalol, and amlodipine). Acute psychosis in the setting of uncontrolled hypertension, hypokalemia, metabolic alkalosis, and venous thromboembolism raised the suspicion for metabolic disorders, such as hypercortisolism, hyperaldosteronism, or pheochromocytoma, as differentials. 

**Figure 3:**
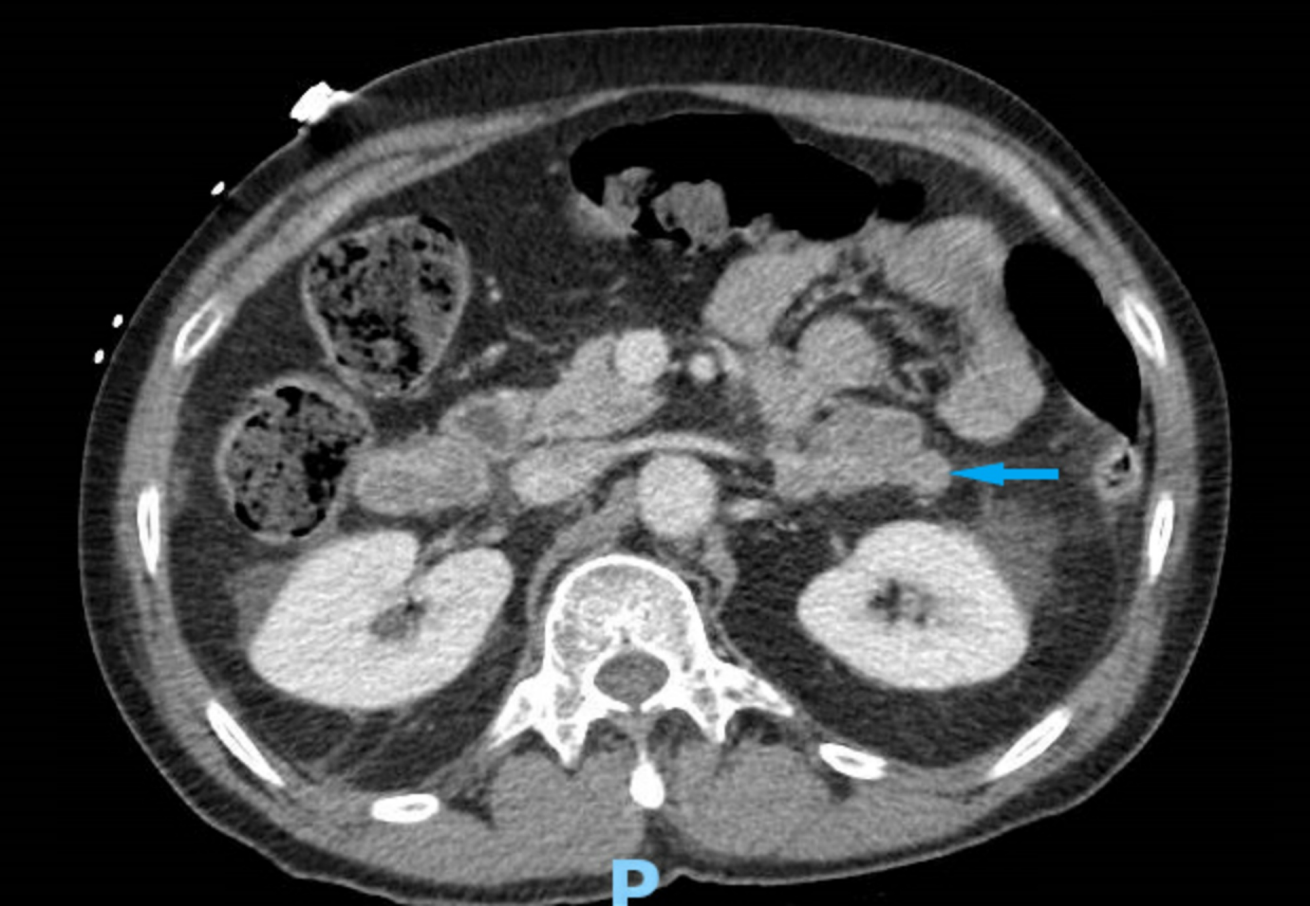
Computed tomography of the abdomen and pelvis showing bilateral adrenal nodules

An endocrinologist was consulted and further workup was initiated. Serum aldosterone was 3.1 (normal: ≤ 23.2 ng/dl) and plasma renin activity was < 2.1 (normal: ≤ 33.2 pg/ml), which ruled out hyperaldosteronism. Plasma metanephrines (< 10; normal: 0 - 62 pg/ml) and normetanephrines (< 10; normal: 0 - 145 pg/ml) were within normal limits. The 24-hour metanephrines (129; normal: < 400 mcg/24 hr), normetanephrines (381; normal: < 900 mcg/24 hr), vanillylmandelic acid (3.9; normal: < 8 mg/24 hr), dopamine (189; normal: 0 - 510 mcg/24 hr), norepinephrine (124; normal: 0 - 135 mcg/24 hr), and epinephrine (9; normal: 0 - 20 mcg/24 hr) were also within normal limits, which ruled out pheochromocytoma. This raised the suspicion of hypercortisolemia. A low-dose dexamethasone suppression test showed inappropriate suppression of plasma cortisol (plasma AM cortisol after overnight 1 mg dexamethasone was 38.4; > 1.8 mcg/dL is abnormal). His serum AM cortisol was 37.6 (normal: 6.2 - 19.4 mcg/dL). The serum ACTH was less than 5 (normal: 0 - 46 pg/ml) on two separate occasions. The 24-hour urinary cortisol was 1,116 (normal: 3.5 - 4.5 mcg/24 hr). This constellation of laboratory findings and the elevated cortisol with low ACTH suggested ACTH-independent CS. A CT scan of the abdomen and pelvis with intravenous contrast was performed to evaluate the adrenal gland which showed multiple nonspecific adrenal nodules bilaterally with the largest one ranging between 3.5 - 4.5 cm bilaterally (Figure 3). The patient was hence diagnosed with ACTH-independent CS in the setting of primary bilateral macronodular adrenal hyperplasia (PBMAH).

The patient was referred for surgery of the adrenal masses, which he refused at the time and opted for non-invasive medical management. He was then started on ketoconazole, 200 mg two times a day, as a bridge to the eventual need for surgical intervention. Over the course of the next two weeks, all his psychiatric symptoms improved significantly. After a week of therapy with ketoconazole, his 24-hour urine cortisol decreased to 589 mcg/24 hr. The patient was also started on spironolactone which improved his hypertension and hypokalemia. With these therapeutic interventions, the patient was able to return to his functional status, requiring only minimal assistance. There was also an improvement in his blood pressure, electrolyte abnormalities, and glycemic control. The patient was followed up on an outpatient basis up to one to two years after discharge from the hospital. His urinary cortisol returned to normal limits and the hemoglobin A1c improved from 8 to 6 after one year of treatment.

## Discussion

Primary adrenal disorders account for 20% - 30% of cases of ACTH-independent CS [1]. Functional unilateral adrenal adenoma or carcinoma constitutes the majority of the cases, while bilateral lesions occur in 10% of cases and include primary pigmented nodular adrenocortical disease (PPNAD), primary bilateral adrenocortical macronodular hyperplasia (BMAH), isolated micronodular adrenocortical disease (iMAD), and rarely, bilateral adenoma or cancer [11]. 

PBMAH is a rare cause (< 2%) of endogenous CS which usually manifests in the fifth and sixth decades of life and is distributed equally amongst both genders [5]. It is characterized by enlarged adrenal glands containing multiple non-pigmented nodules > 10 mm in diameter. The pathophysiology of hypercortisolemia in PBMAH has been evolving. It was previously known to be an ACTH-independent macronodular adrenal hyperplasia; however, several recent studies have shown that cortisol secretion in BMAH is partially regulated by intra-adrenal ACTH, which is mainly produced by steroidogenic cells in the hyperplastic adrenals [12-13]. Thus, the cortisol production in PBMAH could partially be ACTH-dependent. Glucocorticoid excess in PBMAH is also reported to be due to aberrant expression of certain receptors in adrenocortical cells which result in unregulated steroidogenesis and hormone excess. This mainly includes receptors for the gastric inhibitory polypeptide, beta-adrenergic receptor agonists, vasopressin (V1-V3R), 5-hydroxytryptamine (5-HT), angiotensin II (AT), glucagon, luteinizing hormone (LH)/human chorionic gonadotropin (hCG), and leptin [14-15]. Diagnosis either occurs as adrenal incidentalomas with no or minimal cortisol overproduction, also identified as subclinical CS, or as a full-blown CS with elevated cortisol and suppressed ACTH with confirmation of adrenal hyperplasia later with radiographic imaging. Common features of CS include centripetal fat deposition, abdominal striae, facial plethora, muscle atrophy, bone density loss, immunosuppression, and cardiovascular complications. Hypokalemia in CS is likely from cortisol excess which has mineralocorticoid activity by saturating the 11 beta-hydroxysteroid dehydrogenase enzyme at the renal tubule. Psychiatric complications include irritability, anxiety, depression, and cognitive impairment [16]. Mania and hypomania are less commonly reported symptoms. Psychosis is a rare manifestation of CS [16]. If underlying hypercortisolism is not treated, psychosis may remain refractory to psychotropic medications. 

Our patient had severe psychosis, uncontrolled diabetes, resistant hypertension, and hypokalemia as possible manifestations of CS. No other signs or symptoms were noted on physical examination. The patient had a very extensive course of neuropsychiatric symptoms which were difficult to control even on multiple psychotropic medications and improved only after the underlying cause was treated. Only on very few occasions has psychosis preceded the diagnosis of CS as happened in our patient [17]. Even out of that, most cases in the literature were secondary to ACTH-dependent Cushing’s disease from a pituitary mass and resolved after transsphenoidal surgery. No cases have been reported where psychosis occurred as a primary manifestation in the diagnosis of CS from PBMAH. Bilateral adrenalectomy is generally recommended for severe hypercortisolism such as in our case. However, our patient refused surgery. Hence, he was managed medically with ketoconazole and later mifepristone on an outpatient basis which also improved the psychiatric symptoms and resistant hypertension. The patient did have relapses of psychotic symptoms and hospitalizations in subsequent years as surgery, which is the definitive treatment for this condition, was not performed. 

Overall, PBMAH treatment depends on the severity of the cortisol excess. Bilateral adrenalectomy is indicated in patients with severe CS (urinary free cortisol > 3 times above the upper normal limit) with no aberrant receptors. Unilateral adrenalectomy is considered in patients with moderate excess in cortisol (< 3 times the upper normal limit of urinary cortisol) with symptoms of CS. Adrenal vein sampling for the selection of which adrenal to be removed is not routinely indicated as standardized data is lacking for the same. Conservative management is considered in mild cortisol excess (normal urinary free cortisol with normal ACTH) and no symptoms of CS with biannual to annual follow-up with biochemical testing. In patients who refuse surgical treatment, ketoconazole is an effective option that controls hypertension, hypokalemia, and diabetes in CS, as it did in our patient. It decreases cortisol in patients with CS and may prevent adrenal overgrowth [18].

## Conclusions

Psychosis is a rare clinical manifestation of CS. Delay in the identification of the cause of psychosis and subsequent treatment can lead to significant morbidity in patients. It often subjects them to unnecessary psychotropic medications and their side effects and affects their overall quality of life. Clinicians should be mindful of considering CS as an important differential in refractory psychosis, especially in the presence of other bodily clues to the disease. PBMAH is a unique entity, mostly requiring surgery as the treatment of choice with few acceptable medical measures to control symptoms. We hereby present an extremely rare case of severe psychosis due to CS with underlying PBMAH. 
